# *Ginkgo biloba* Exocarp Extract Inhibits the Metastasis of B16-F10 Melanoma Involving PI3K/Akt/NF-*κ*B/MMP-9 Signaling Pathway

**DOI:** 10.1155/2018/4969028

**Published:** 2018-06-25

**Authors:** Chenjie Cao, Ya Su, Yanqi Gao, Chengrong Luo, Lu Yin, Yingjie Zhao, Huasheng Chen, Aihua Xu

**Affiliations:** ^1^Department of Pharmacology, Medical College of Yangzhou University, Yangzhou, Jiangsu Province 225001, China; ^2^Department of Combination of Traditional Chinese and Western Medicine, Medical College of Yangzhou University, Yangzhou, Jiangsu Province 225001, China; ^3^Jiangsu Co-Innovation Center for Prevention and Control of Important Animal Infectious Diseases and Zoonoses, Yangzhou, Jiangsu Province 225001, China

## Abstract

In recent years, interest in natural plant extracts for cancer treatment is growing in the drug development field.* Ginkgo biloba* exocarp extract (GBEE) is known for possessing inhibitory effects on various mouse and human cancer cells. And no adverse reactions were observed during its clinical application to cancer patients. The aim of this study is to investigate the inhibitory effect of GBEE on the metastasis of B16-F10 melanoma and its related mechanisms. The B16-F10 melanoma lung metastasis model was established in C57BL/6J mice. It was found that GBEE inhibited the growth and pulmonary metastasis of B16-F10 melanoma transplanted tumor and downregulated the level of MMP-9 protein. Meanwhile, the B16-F10 cells were used to study in vitro. The results showed that GBEE inhibited the proliferation and migration of B16-F10 cells. Simultaneously, it suppressed the heterogeneous adhesion of B16-F10 cells to human umbilical vein endothelial cells (HUVEC) in a concentration-dependent manner. In addition, the levels of p-PI3K, p-Akt, NF-*κ*B, and MMP-9 were decreased, while the PI3K and Akt were not significantly changed. These results indicate that GBEE can inhibit the metastasis of B16-F10 melanoma via multiple links and the molecular mechanism involved the regulation of PI3K/Akt/NF-*κ*B/MMP-9 signaling pathway.

## 1. Introduction

Malignant melanoma (MM) is a kind of cancer with high metastatic potential, which is formed by the malignant transformation of normal melanocytes [1]. It is one of the most rapidly growing malignancies in the world with an annual increase of 3%-5% [2].

At present, the clinical commonly used chemotherapy drugs such as dacarbazine and temozolomide have a certain therapeutic effect, but it often occurs that melanoma cells metastasize and spread during chemotherapy in patients, which is the main reason leading to the short survival and poor prognosis [3, 4]. Therefore, the control of the metastasis of melanoma has become the key to the whole treatment [5]. In recent years, many studies have found that natural products have good therapeutic effects on cancer [6, 7], and some extracts of traditional Chinese medicine can inhibit the metastasis of malignancy [8, 9].

*Ginkgo biloba* L. is also known as Ginkgo nuts. It is a unique tree species in China, which is rare and ancient. Both the leaves and fruits have important medicinal value [10].* Ginkgo biloba *exocarp is the succulent skin outside Ginkgo nuts.* Ginkgo biloba *exocarp extract (GBEE) is a mixture extracted from* Ginkgo biloba *exocarp using water solution-alcohol precipitation method according to the process of Chinese invention patent in our laboratory, taking proteoglycan as the main active ingredient [11]. In addition, it does not contain the harmful ingredient, ginkgolic acid [12]. Previous studies have shown that GBEE has inhibitory effects on various mouse and human cancer cells [13–15], and it can also promote the immune function of the body [16, 17]. Meanwhile, no adverse reactions were observed during its clinical application to cancer patients. It has good application prospects [18, 19]. Studies on GBEE inhibiting the metastasis of melanoma have not yet been involved. In this study, the B16-F10 melanoma cell line was selected. The inhibitory effect of GBEE on B16-F10 melanoma metastasis and its related mechanisms were explored in vivo and in vitro by establishing B16-F10 melanoma lung metastasis model in mice and culturing B16-F10 cells in vitro.

## 2. Materials and Methods

### 2.1. GBEE

Ginkgo nuts samples were obtained from Taixing (Jiangsu Province, China), identified by Meng Yin (Director of pharmacists) in Yangzhou Food and Drug Inspection and Testing Center (Jiangsu Province, China) as the family plant of* Ginkgo biloba* L. The succulent skin was peeled off by hand. The GBEE was prepared according to the invention patent method in our laboratory (Patent Number: CN 201010251050.9) [11]. The final concentration of ethanol was 80%, and the precipitate was dried in vacuo. Then the off-white powder was obtained and sealed at room temperature. The content of proteoglycan was 66.4%, measured by phenol-sulfuric acid method and brilliant blue method. Infrared Spectroscopy (IR) reported that the GBEE contained characteristic peak of polysaccharide. High Performance Liquid Chromatography (HPLC) and Thin Layer Chromatography (TLC) made the fact that the polysaccharide contained 7 kinds of monosaccharides including mannose, rhamnose, galacturonic acid, glucose, galactose, fructopyranose, and arabinose clear. The protein contained 14 kinds of amino acids including aspartic acid, glutamic acid, serine, glycine, threonine, alanine, proline, valine, methionine, isoleucine, leucine, phenylalanine, tryptophan, and lysine [20]. The voucher specimen of GBEE was deposited at the pharmacy experimental center in Medical College of Yangzhou University.

### 2.2. Chemicals and Reagents

Dulbecco's modified eagle medium (DMEM) and fetal bovine serum (FBS) were obtained from Gibco (Grand Island, NY, USA). Penicillin and streptomycin were purchased from Lukang Pharmaceutical (Shandong, People's Republic of China). cis-Dichlorodiammineplatinum (II) (DDP) was purchased from Jingkehongda Biotechnology (Beijing, People's Republic of China). 3-(4, 5-Dimethylthiazol-2-yl)-2, 5-diphenyl-tetrazolium bromide (MTT) was purchased from Sigma-Aldrich (St. Louis, MO, USA). Tris base, glycine, sodium dodecyl sulphate (SDS), Tween 20, Bovine Serum Albumin (BSA), and polyvinylidene fluoride (PVDF) membrane were obtained from Biosharp (Anhui, People's Republic of China). Sodium chloride (NaCl), ethanol, methanol, and other chemical reagents were derived from Sinopharm Chemical Reagent (Beijing, People's Republic of China). Immunohistochemistry kit, Radio-Immunoprecipitation Assay (RIPA) lysate, and 3,3′-diaminobenzidine (DAB) were obtained from Boster (Wuhan, People's Republic of China). Trizol, diethyl pyrocarbonate (DEPC), primers, M-MuLV First Strand cDNA Synthesis Kit, 2xSG Fast qPCR Master Mix, and secondary antibody consisting of horseradish peroxidase-conjugated IgG were purchased from BBI (Shanghai, People's Republic of China). BeyoECL Plus and Enhanced BCA Protein Assay Kit were purchased from Beyotime (Shanghai, People's Republic of China). NF-*κ*B rabbit anti-mouse antibody was obtained from Wanleibio (Shenyang, People's Republic of China). Phosphor-PI3K (p-PI3K), PI3K, phosphor-Akt (p-Akt), and Akt anti-mouse rabbit antibodies were purchased from Cell Signaling Technology (MA, USA). GAPDH and MMP-9 anti-mouse rabbit antibodies were obtained from Abcam (Cambridge, England).

### 2.3. Cell Culture

The human umbilical vein endothelial cell (HUVEC) line and B16-F10 cell line were purchased from Shanghai cell bank, Chinese Academy of Sciences. The cells were cultured in DMEM with 10% FBS, 100 U/mL penicillin, and 100 g/ml streptomycin. The cells were incubated in a humidified incubator at 37°C with 5% CO_2_ (v/v) and subcultured according to trypsin digestion method. The passage time was usually 3 days.

### 2.4. Animals

The C_57_BL/6J mice were provided by the Center of Comparative Medicine of Yangzhou University. The mice (SPF class) were female and 6 weeks old, weighing 18-22 g. Animal Certificate was SCXK Su 2012-0004; animal use license was SYXK Su 2012-0029. They had been acclimated on 12 h light/12 h dark cycle for one week before use. All the animal studies were conducted in conformity with the Institutional Animal Ethics Committee of Yangzhou University and the National Institutes of Health Guide for Care and Use of Laboratory Animals.

### 2.5. Detection of the Tumor Inhibition Rate and Antipulmonary Metastasis Rate

The B16-F10 cells were diluted by normal saline, and the cell suspension (1.0×10^7^ cells/mL) was prepared. A volume of 0.2 mL of such B16-F10 cell suspension was inoculated subcutaneously in the right forelimb armpit of the mice. Once the transplanted tumor grew to appropriate size, the tumor tissue was removed and cut. The tumor cell suspension was prepared according to the conventional methods. The density was adjusted to 1.0×10^7^ cells/mL and the cell suspension was inoculated subcutaneously in the right forelimb armpit of the mice. Each mouse was injected with a volume of 0.2 mL tumor cell suspension. 24 h after inoculating, the mice were randomly divided into 6 groups, and there were ten mice in each group. Groups and dose: the normal control group (NCG, without tumor cells) and the model control group (MCG) were given normal saline [0.1 mL/10 g (body weight)] by intragastric gavage (ig), once a day for 17 days; the mice in positive control group (PCG) were given DDP [5 mg/kg (body weight)] by intraperitoneal (ip), every other day for 7 days; the GBEE groups were given GBEE [50, 100, 200 mg /kg (body weight)] by ig, once a day for 17 days. On the 18th day, the mice were sacrificed by cervical dislocation and the transplanted solid tumor was completely stripped and weighed. The tumor inhibition rate = (average tumor weight in MCG - average tumor weight in treated groups)/average tumor weight in MCG ×100%.

The lung tissue were removed and fixed. The number of lung metastasis foci was counted and the diameter was gauged, using anatomical microscope (Nikon, Tokyo, Japan). The number of metastasis foci = I ×1 +II×2 +III×3 +IV×4 (according to the diameter, the foci are divided into four levels: Level I < 0.5 mm, 0.5 mm ≤ Level II < l mm, l mm≤ Level III ≤ 2 mm, Level IV > 2 mm). The antimetastatic rate (%) = (average number of lung metastasis foci in MCG - average number of lung metastasis foci in treated groups)/average number of lung metastasis foci in MCG × 100%.

### 2.6. Hematoxylin-Eosin (HE) Staining Assay

The lung tissues were fixed in 10% neutral formalin for 24 h, then dehydrated with ethanol, and transparent with xylene. They were embedded in paraffin and cut into slices. The dried slices were transparent with xylene for 5-10 min and then moved into the hematoxylin to soak and stain for 1-2 min. Then they were differentiated in 1% hydrochloric acid alcohol and stained using eosin for 2-5 min. Finally, the slices were dehydrated with alcohol, transparent with xylene, and sealed in resin. Then the pathological slices of lung tissue were observed using optical microscope (Nikon, Tokyo, Japan). The B16-F10 cells are characterized by large nuclei, deep staining, and marked atypia; simultaneously, they accumulated as clumps whose shape and distribution were irregular.

### 2.7. Immunohistochemistry Assay

The transplanted tumor tissues were fixed in 10% neutral formalin for 24 h and then dehydrated with ethanol and transparent with xylene. They were embedded in paraffin and cut into slices. The dried slices were transparent with xylene and dehydrated with gradient of ethanol. The activity of endogenous peroxidase on sections was blocked with 3% H_2_O_2_. The antigens were repaired with microwave ovens. In order to reduce nonspecific reactions, the slices were blocked by 5% BSA before incubated with MMP-9 antibody overnight. Then the slices were incubated with horseradish peroxidase-conjugated Goat Anti-Rabbit IgG and stained with DAB and SABC. The slices were finally counterstained with hematoxylin. Phosphate buffer saline (PBS) was used to wash the slices after each step and replace the primary antibody as a negative control. The results of the target protein were obtained under optical microscope (Nikon, Tokyo, Japan). The expression of MMP-9 protein was located in the cytoplasm and the positive staining showed brown. Each slice was randomly selected five fields in the high magnification, and the mean integrated optical density (IOD) of MMP-9 positive chromatin was determined using image analysis software Image-pro Plus 6.0.

### 2.8. MTT Assay

The B16-F10 cells in logarithmic growth phase (2×10^5^ cells/mL) were seeded in 96-well plate. After cell attachment, the treated groups were given DMEM medium containing GBEE, and the final concentrations of GBEE were 5, 10, 20, 40, 80, 160, and 320 *μ*g/mL. The cells in PCG were treated with DDP (5 *μ*g/mL) and the cells in blank control group were treated with equal volume of DMEM medium. MTT solution (5 mg/mL) was added after the cells were cultured in a humidified atmosphere of 5 % CO_2_ at 37°C for 44 h. Subsequently, the cells were incubated for 4 h. Then the culture medium was discarded and the acidified isopropanol was added. After the blue purple crystal fully dissolved, the optical density (OD) was measured using a microplate reader (MULTISKAN FC, Thermo, MA, USA) at 570 nm. Finally, the inhibition rate was calculated pursuant to the following formula: the inhibition rate= (1- OD _treated  groups_/ OD _blank  control  group_) ×100%.

### 2.9. Wound Healing Assay

The B16-F10 cells in logarithmic growth phase were digested by 0.25% pancreatin and were evenly placed on 6-well plate (1×10^6^ cells/mL). After the cells were grown to 90% confluence, the center of each cell monolayer was scraped with a sterile 200 *μ*L tip pipette as to create a denuded zone. The DMEM medium was discarded, and each well was washed twice. Then the cells were treated with GBEE (10, 20, and 40 *μ*g/mL) and DDP for 48h. During this period, the changes in the migration of the cells were observed and photographed using inverted microscope (Nikon, Tokyo, Japan) every 12 h. The area and height of the wound were examined using Image-pro Plus 6.0. The area divided by the height gives the average width of the wound. Cell migration rate= (the average width at 0 h - the average width at 12, 24, 36 or 48 h) / the average width at 0 h) ×100%.

### 2.10. Cell Adhesion Assay

The HUVEC were the target cells in this assay, and the B16-F10 cells were the tested cells. The B16-F10 cells (2×10^5^/mL) in logarithmic growth phase were inoculated into the culture flasks. After 12 h of cultivation, GBEE (10, 20, 40 *μ*g/mL) and DDP were added (1mL/culture flask). And the cells in blank control group were treated with equal volume of DMEM medium. They were all treated for 24 h.

The HUVEC were seeded in 96-well plate (100 *μ*L/well) at a density of 2×10^5^ cells/mL and then cultured for 12 h. When the target cells grew to confluence, the B16-F10 cells that have been treated for 24 h were collected and seeded into the target cells at a density of 2×10^5^ cells/mL (100 *μ*L/well). Subsequently, the two kinds of cells were cocultured for 2 h, making the tested cells fully adhered to the target cells. After the cultivation was completed, the nonadherent cells were removed. MTT method was applied to detect the OD value. The cell adhesion inhibition rate = (1- OD_treated  groups_/ OD_blank  control  group_) ×100%.

### 2.11. qRT-PCR Assay

The B16-F10 cells (2×10^5^ cells/mL) in logarithmic growth phase were seeded in 6-well plate. GBEE (10, 20, 40 *μ*g/mL) and DDP were added after cell adherence, and the blank control group were established. The total RNA was extracted with Trizol (300 *μ*L/well) after the cells were treated for 48 h. The RNA was reverse transcribed into cDNA using M-MuLV First Strand cDNA Synthesis Kit. Then the cDNA was amplified with 2xSG Fast qPCR Master Mix using PCR instrument (Roche Light Cycler 96, Basel, Switzerland). GAPDH was the reference gene. The sequences of primers are shown in Table 1. Each sample was repeated three times and calculated the average cycle threshold (Ct). The real-time fluorescence quantitative was analyzed by 2^−ΔΔCt^ method, and the value of RQ (relative quantification) was calculated.

### 2.12. Western Blot Assay

The B16-F10 cells (2×10^5^ cells/mL) in logarithmic growth phase were seeded in 6-well plate. The cells were treated in the same way as that in “Section 2.11”. The total protein was extracted after the cells were treated for 48 h, and the concentration was determined by BCA assay. The total protein was separated by SDS polyacrylamide gel electrophoresis and then transferred to PVDF membrane. 5% BSA was used to seal the membrane at room temperature for 1.5 h. The membrane was incubated with the primary antibody overnight at 4°C and the secondary antibodies conjugated to horseradish peroxidase were added. After 4 h, the fluorescent substrate was prepared and the results were recorded in gel imaging system (BIO-RAD, Hercules, CA, USA). The analyzing software (Image J) was used to analyze the gray value. The relative levels of target proteins were obtained, taking GAPDH as the internal reference.

### 2.13. Statistical Analysis

All data in this experiment were analyzed using SPSS 17.0 (SPSS Inc., Chicago, IL, USA) and presented as mean ± standard deviation (SD). The groups were compared using F test and Dunnett's t-test.* P* <0.05 was considered to be significantly different.

## 3. Results

### 3.1. GBEE Inhibited the Growth and Metastasis of B16-F10 Melanoma Transplanted Tumor

**Figure 1:**
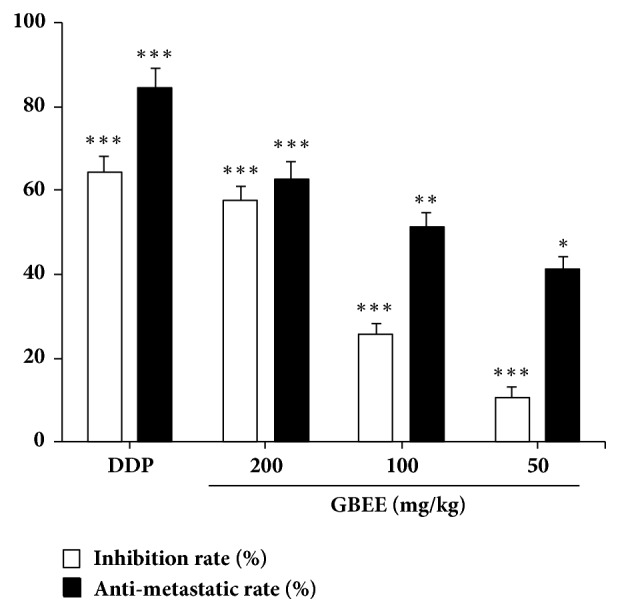
GBEE inhibited the growth and pulmonary metastasis of B16-F10 melanoma transplanted tumor. Mice with transplantation tumor were treated with NS, DDP, and GBEE (50, 100, and 200 mg/kg). After giving corresponding drugs for 17 days, the tumor blocks and lung tissues were stripped, and the inhibition rate and antimetastatic rate were calculated. Data are shown as mean ± SD (*n *= 10). ^*∗*^*P*<0.05, ^*∗∗*^*P*<0.01, and ^*∗∗∗*^*P*<0.001 versus model.

GBEE (50, 100 and 200mg/kg) had a significant inhibitory effect on the growth of B16-F10 transplanted tumor with a dose-effect relationship. Simultaneously, the antipulmonary metastasis rate was increased with the increase of dose, appearing a significant antimetastasis effect (Figure 1).

### 3.2. Pathological Observation of Lung Metastasis Foci

**Figure 2:**
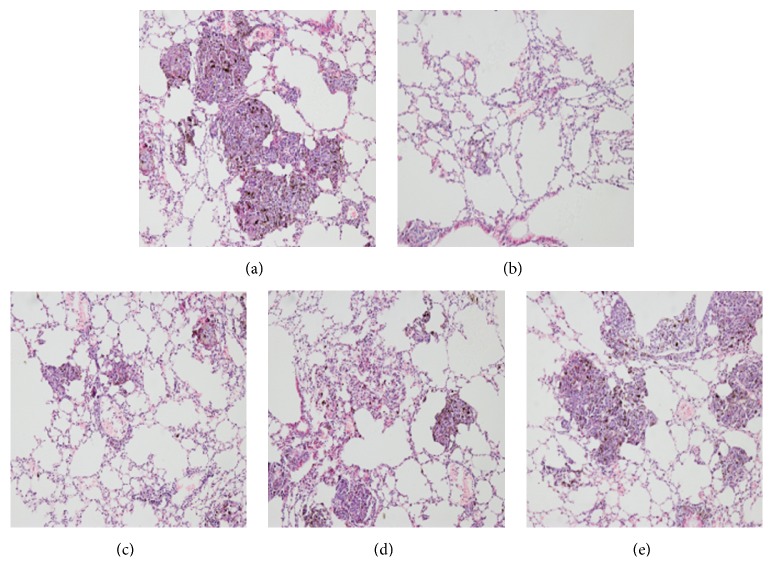
Histological examination of lung tissue and antimetastatic rate in different groups. Mice with transplantation tumor were treated with (a) NS, (b) DDP, GBEE (c) 200mg/kg, (d) 100 mg/kg, and (e) 50 mg/kg. After giving corresponding drugs for 17 days, HE staining analysis was carried out. The nucleus of cancer cells in metastasis foci was dyed blue and the cytoplasm was dyed red (100×).

HE staining revealed that the number and area of lung metastases foci were both significantly reduced in all GBEE (50, 100, and 200 mg/kg) treated groups compared with the model group, and the effect of high dose group was more obvious (Figure 2).

### 3.3. GBEE Inhibited the Level of MMP-9 Protein in Transplanted Tumor

**Figure 3:**
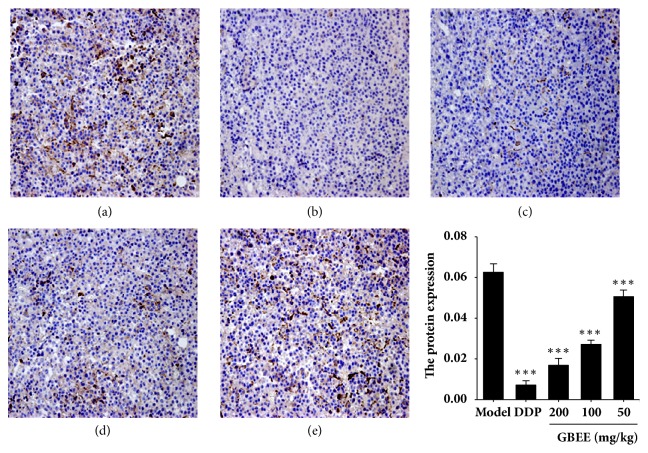
GBEE inhibited the level of MMP-9 protein in transplanted tumor. Mice with transplantation tumor were treated with (a) NS, (b) DDP, GBEE (c) 200mg/kg, (d) 100mg/kg, and (e) 50 mg/kg. After giving corresponding drugs for 17 days, the immunohistochemistry analysis was carried out (200×). The average IOD of positive staining of MMP-9 was measured by Image-pro Plus 6.0 software. Data are shown as mean ± SD (*n *= 3). ^*∗∗∗*^*P*<0.001 versus model.

Compared with the model group, the protein levels of MMP-9 in transplanted tumor were decreased with a dose-effect relationship in the GBEE (50, 100, and 200 mg/kg) groups (Figure 3).

### 3.4. GBEE Inhibited the Proliferation of B16-F10 Cells In Vitro

**Figure 4:**
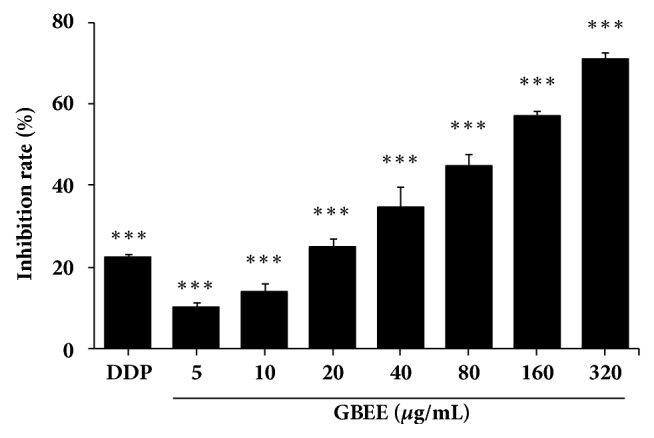
GBEE inhibited the proliferation of B16-F10 cells in vitro. The B16-F10 cells were treated with DDP and GBEE for 48 h. MTT assay was carried out. Data are shown as mean ± SD (*n *= 6). ^*∗∗∗*^*P*<0.001 versus control.

The results of MTT assay showed that after GBEE (5-320 *μ*g/mL) treated the B16-F10 cells for 48 h, the proliferation of the cells was inhibited in a concentration-dependent manner. The half maximal inhibitory concentration (IC50) value was 94.0 *μ*g/mL (Figure 4).

### 3.5. GBEE Inhibited the Migration of B16-F10 Cells

**Figure 5:**
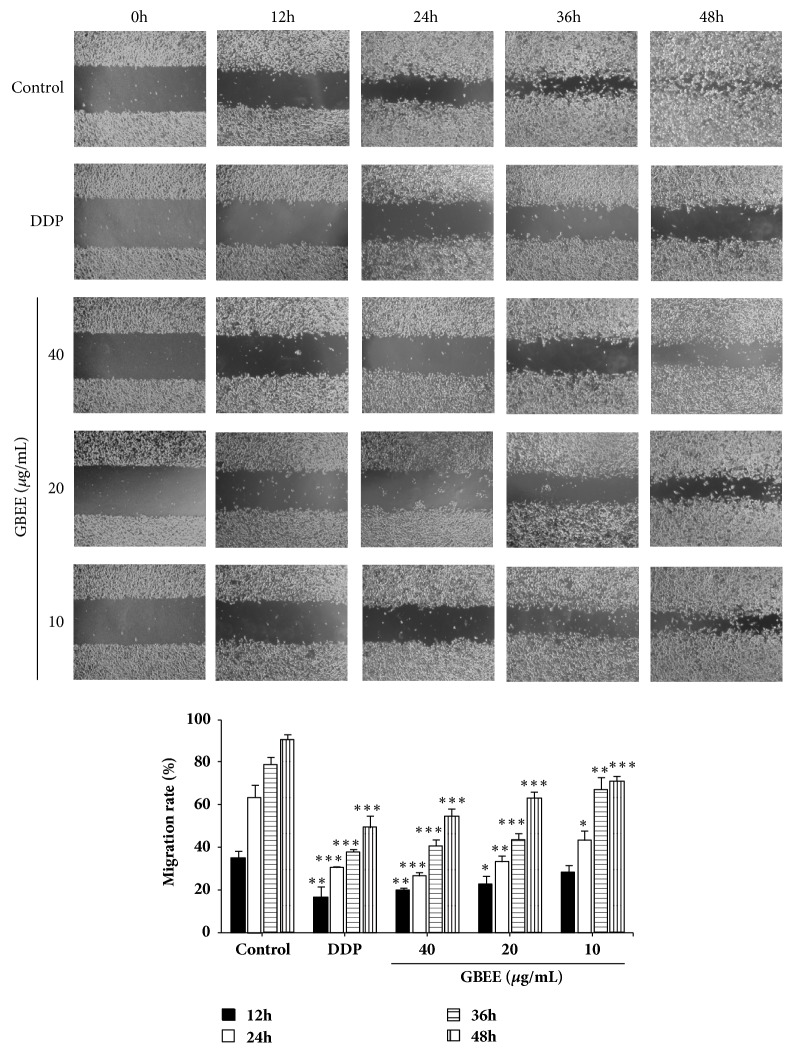
GBEE inhibited the migration of B16-F10 cells. The B16-F10 cells were treated with blank DMEM, DDP, and GBEE (10, 20, and 40 *μ*g/mL) for 48h. The migration of cells was observed. The cell mobility was measured by Image-pro Plus 6.0 software. Data are shown as mean ± SD (*n *= 3). ^*∗*^*P*<0.05, ^*∗∗*^*P*<0.01, and ^*∗∗∗*^*P*<0.001 versus control.

Wound healing assay explained that the cells in each group had no significant difference at 0 h. After culturing for 48 h, the wound in control group was almost fully healed, whereas the speed of wound healing in GBEE (10, 20, and 40 *μ*g/mL) treated groups was significantly slowed down. Furthermore, compared with the control group, the inhibitory effect of GBEE on the migration of B16-F10 cells was dose-dependent as well as time-dependent (Figure 5).

### 3.6. GBEE Inhibited the Adhesion of B16-F10 Cells to HUVEC

**Figure 6:**
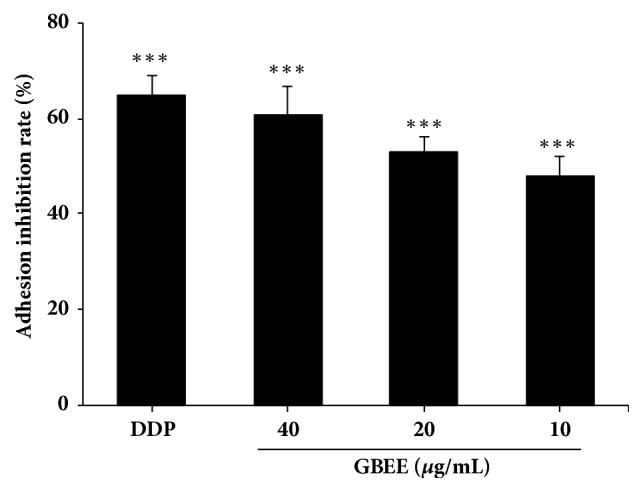
GBEE inhibited the adhesion of B16-F10 cells to HUVEC. The B16-F10 cells were treated with blank DMEM, DDP, and GBEE (10, 20, and 40 *μ*g/mL) for 48 h. Heterogeneous adhesion experiment was carried out. Data are shown as mean ± SD (*n *= 6). ^*∗∗∗*^*P*<0.001 versus control.

As shown in Figure 6, GBEE (10, 20, and 40 *μ*g/mL) significantly inhibited the heterogeneous adhesion of B16-F10 cells to HUVEC. The inhibitory effects were gradually increased as the concentration of GBEE increased.

### 3.7. GBEE Inhibited PI3K/Akt/NF-*κ*B/MMP-9 Signaling Pathway in B16-F10 Cells

**Figure 7:**
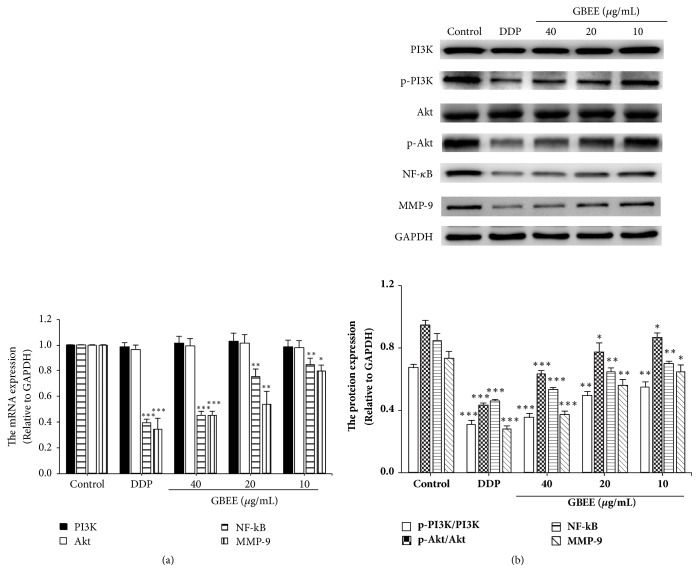
GBEE inhibited PI3K/Akt/NF-*κ*B/MMP-9 signaling pathway in B16-F10 cells. The B16-F10 cells were treated with blank DMEM, DDP, and GBEE (10, 20, 40 *μ*g/mL) for 48 h. (a) The mRNA levels of NF-*κ*B, MMP-9, PI3K, and Akt in cells were analyzed by qRT-PCR assay. (b) The protein levels of p-PI3K, PI3K, p-Akt, Akt, NF-*κ*B, and MMP-9 were determined by Western Blot assay. Data are shown as mean ± SD (n = 3). ^*∗*^*P*<0.05, ^*∗∗*^*P*<0.01, and ^*∗∗∗*^*P*<0.001 versus control.

qRT-PCR results showed that GBEE (10, 20, and 40 *μ*g/mL) downregulated the mRNA levels of NF-*κ*B and MMP-9 in a concentration-dependent manner and had no significant effect on the mRNA level of PI3K and Akt (Figure 7(a)).

Western Blot results explained that GBEE suppressed the protein levels of p-PI3K, p-Akt, NF-*κ*B, and MMP-9 in B16-F10 cells in a concentration-dependent manner and had no significant effect on the PI3K and Akt protein (Figure 7(b)).

## 4. Discussion

For the first time, the study found that GBEE (50-200 mg/kg) could inhibit the growth of B16-F10 melanoma transplanted tumor and had an antipulmonary metastatic effect. HE staining showed that GBEE significantly reduced the number and area of lung metastases foci. These suggested that GBEE has an inhibitory effect on the metastasis of B16-F10 melanoma. Based on this, further research was conducted in this paper.

Tumor metastasis is a dynamic, continuous, and complex process. After the occurrence of malignant tumor, the overproliferation of primary tumor cells accelerates the growth of tumor tissue, which in turn causes the internal pressure of tumor tissues to rise, so that the tumor cells can be separated from the primary tumor foci. After that, the tumor cells invade the basement membrane (BM), and by means of secreting the related factors, the protein of BM is cracking and the gaps are formed, and then the tumor cells can move through the extracellular matrix (ECM) via the gap. Afterwards, the tumor cells adhere to the endothelial cells of the local capillaries or lymphatic capillaries and penetrate the wall, so that the tumor cells can reach the tissues and organs of the whole body with blood or lymph and continue to proliferate and form metastasis foci [21, 22]. Thus, the tumor cell proliferation, migration, heterogeneous adhesion, and the degradation of ECM and BM are all the key steps in tumor metastasis. MMP-9 is a kind of matrix metalloproteinases (MMPs). It has increased expression and activity in metastatic tumors and can specifically degrade ECM and BM [23, 24]. The results of immunocytochemistry showed that GBEE (50-200 mg/kg) significantly reduced the expression of MMP-9 protein with a dose-effect relationship in the transplanted tumor. The in vitro results showed that GBEE (5-320 *μ*g/mL) inhibited the proliferation of B16-F10 melanoma cells in a concentration-dependent manner. GBEE also had inhibitory effects on the migration of B16-F10 cells and the heterogeneous adhesion of B16-F10 to HUVEC in a concentration-dependent manner at the concentration of 10-40 *μ*g/mL. These results suggested that the antimetastasis effect of GBEE on B16-F10 melanoma is related to its inhibition of tumor cell proliferation, migration, heterogeneous adhesion, and ECM and BM degradation.

NF-*κ*B is an important nuclear transcription factor that participates in regulating metastasis and many other actions of tumor cells [25]. In the resting state, NF-*κ*B combines with its endogenous inhibitory factor I*κ*B and forms a complex, so that it exists in the cytoplasm in an inactive state [26]. Studies have found that the upstream signaling factors in tumor cells can activate I*κ*B, making it dissociate from the complex. Then NF-*κ*B will be activated and enter the nucleus [27]. The promoter region of MMP-9 contains the binding site of NF-*κ*B. So the activation of NF-*κ*B can increase the transcriptional activity of MMP-9, thereby the level of MMP-9 is promoting [28, 29]. As a result, inhibiting the activation of NF-*κ*B can decrease the level of MMP-9 [30]. As one of the enzymes that specifically degrade ECM and BM, MMP-9 is also closely related to the proliferation, migration, and heterogeneous adhesion of tumor cells. MMP-9 can promote tumor cell proliferation by releasing the cell-membrane-bound precursors of some growth factors [31, 32]. MMP-9 can also promote the tumor cells to move forward by interacting with the integrins on the surface of the cells and modulating its function [33, 34]. Moreover, MMP-9 can enhance the heterogeneous adhesion of tumor cells via activating related cytokines [35] and then increasing the expression of intercellular adhesion molecule [36, 37]. Therefore, inhibiting MMP-9 expression can interfere with the tumor cell proliferation, migration, heterogeneous adhesion, and ECM and BM degradation. Studies have shown that the PI3K/Akt pathway is one of the major pathways that mediates cellular signaling. It participates in multiple processes or links in tumor metastasis [38]. When PI3K/Akt signaling pathway is activated in the tumor cells, the activated Akt activates I*κ*B by phosphorylating I*κ*B kinase. Then NF-*κ*B is activated and enters the nucleus [39, 40]. Scholars have confirmed that the PI3K/Akt signaling pathway can increase the level of MMP-9 through activating the NF-*κ*B; therefore, the metastasis of tumor cells was enhanced [41]. These showed that PI3K/Akt/NF-*κ*B/MMP-9 signaling pathway is involved in the regulation of tumor cell proliferation, migration, heterogeneous adhesion, and ECM and BM degradation. The in vitro results showed that at the mRNA and protein levels, GBEE (10-40 *μ*g/mL) inhibited the expression of p-PI3K, p-Akt, NF-*κ*B, and MMP-9 in a concentration-dependent manner in B16-F10 cells, while it had no significant effect on the PI3K and Akt. These results demonstrated that the inhibitory effect of GBEE on tumor cell proliferation, migration, heterogeneous adhesion, and ECM and BM degradation is related to the regulation of PI3K/Akt/NF-*κ*B/MMP-9 signaling pathway.

## 5. Conclusions

Overall, the mechanisms of the antimetastasis effect of GBEE on B16-F10 melanoma metastasis involve regulating PI3K/Akt/NF-*κ*B/MMP-9 signaling pathway and further inhibiting tumor cell proliferation, migration, heterogeneous adhesion, and ECM and BM degradation.

## Figures and Tables

**Table 1:** List of qRT-PCR primers.

Gene	Forward primer	Reverse primer
PI3K	5′-AACTCTGGGGATGACCTGGA-3′	5′-AGGCGGTCACAACACTCCTA-3′
Akt	5′-AGGGTTGGCTGCACCGCG-3′	5′-GTTGTTGAAGAGACACCGCG-3′
NF-*κ*B	5′-AGTTGAGGGGACTTTCCCAGGC-3′	5′ -TCAACTCCCCTGAAAGGGTCCG-3′
MMP-9	5′-AGACCAAGGGTACAGCCTGTTC-3′	5′-GGCACGCTGGAATGATCTAAG-3′
GAPDH	5′-ACACCCACTCCTCCACCTTT-3′	5′- TAGCCAAAT TCGTTGTCATACC -3′

## Data Availability

The original experimental data used to support the findings of this study were included within the article.
